# Plexiform Schwannoma of the Middle Finger: A Rare Benign Mesenchymal Tumor Presenting as a Chronic Digital Swelling

**DOI:** 10.7759/cureus.94073

**Published:** 2025-10-07

**Authors:** Ashwini Pitambra, Shailaja Prabhala, Shrinivas B Somalwar, Krishna Ramavath

**Affiliations:** 1 Pathology and Laboratory Medicine, All India Institute of Medical Sciences, Bibinagar, Hyderabad, IND; 2 General Surgery, All India Institute of Medical Sciences, Bibinagar, Hyderabad, IND

**Keywords:** histopathology (hp), immunohistochemistry (ihc), peripheral nerve sheath tumours, plexiform schwannoma, rare soft tissue tumor

## Abstract

Plexiform schwannoma (PS) is a rare, benign peripheral nerve sheath tumor characterized by a multinodular growth pattern. While common in superficial locations, such as the dermis and subcutis, its manifestation in the fingers is uncommon and can pose diagnostic challenges. We report the case of a 38-year-old female with a 10-year history of a slow-growing, mildly painful swelling over the proximal phalanx of the right middle finger. Clinical examination suggested a benign soft tissue tumor. The lesion was surgically excised under local anesthesia. Histopathology revealed a predominantly dermal-based tumor, which was unencapsulated and composed of spindle cells with hypocellular and hypercellular areas, consistent with a benign nerve sheath tumor. Immunohistochemistry showed diffuse, strong S-100 positivity, confirming the diagnosis of PS. The postoperative course was uneventful, with no recurrence at the one-year follow-up. This case highlights the importance of including PS in the differential diagnosis of soft tissue tumors of the hand to prevent misdiagnosis and ensure optimal surgical outcomes.

## Introduction

Schwannomas are benign, slow-growing nerve sheath tumors composed of well-differentiated Schwann cells. Histologically, schwannomas demonstrate several histologic subtypes, including cellular, melanotic, conventional, epithelioid, and plexiform variants [1]. Plexiform schwannoma (PS) is a rare subtype, accounting for approximately 5% of all schwannomas and 3-19% of benign soft tissue neoplasms [1,2]. It is defined by a plexiform or multinodular intraneural growth pattern [3,4]. These tumors are usually located in cutaneous or subcutaneous tissues, most commonly in the head, neck, trunk, and flexor aspects of extremities [2,4,5].

Clinically, PS typically presents as a solitary, skin-colored, slow-growing lesion measuring less than 2-4 cm [3,4]. Although most lesions are asymptomatic and discovered incidentally, patients may present with localized pain, paresthesia, or rarely motor deficits due to compression of adjacent nerves [2,5]. MRI and ultrasonography can assist in diagnosis by revealing multiple bead-like nodular structures along the course of the nerve with characteristic signal intensities [6].

Epidemiologically, PS shows no gender predilection and most frequently occurs in the third to sixth decades of life, though cases in childhood and even at birth have been documented [2,3]. While most cases are sporadic, associations with NF2, schwannomatosis, trauma, positive family history, and syndromes such as Gorlin-Koutlas have been reported [4,5].

Due to its intraneural and multinodular nature, managing PS is challenging. Complete surgical excision remains the treatment of choice to minimize recurrence while preserving nerve function [2,4]. Here, we present a rare case of PS arising in the right middle finger of a 38-year-old woman. The lesion was excised entirely, and the diagnosis was confirmed by histopathology and immunohistochemistry. This case contributes to the limited literature on digital PS and underscores the importance of recognizing this entity among soft tissue tumors of the hand.

## Case presentation

Clinical impression and management

A 38-year-old female presented with a 10-year history of a gradually enlarging swelling over the proximal region of the right middle finger. The swelling had increased slowly in size and was associated with mild pain during manual activities. There was no history of trauma, similar swellings elsewhere, or family history of neurocutaneous syndromes.

**Figure 1:**
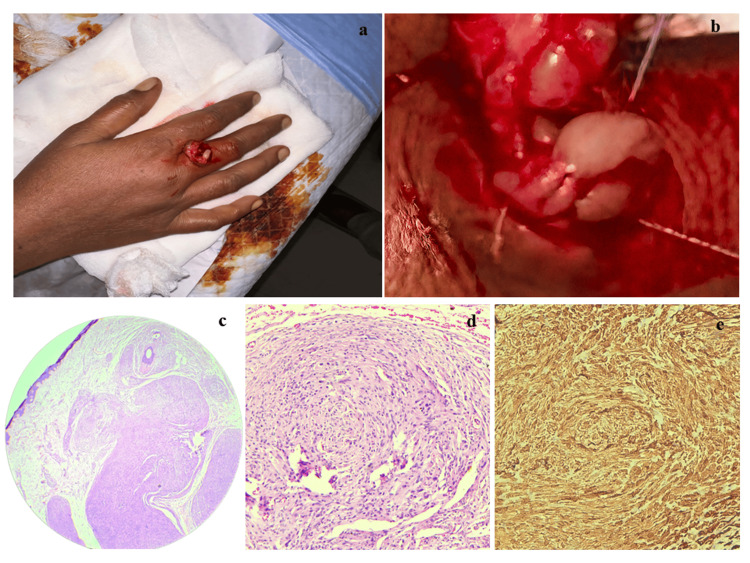
Clinical, histopathological, and immunohistochemical findings 1a: Clinical photograph of the lesion on the right middle finger. 1b: Intraoperative photograph showing a greyish white nodular lesion. 1c: Multinodular lesion in the dermis composed of bland spindle cells in a plexiform pattern (5X, H&E stain). 1d: Nodules with bland spindle cells having wavy nuclei (40X, H&E stain). 1e: Immunohistochemical stain S100, strongly and diffusely positive (40X, IHC stain).

On clinical examination, a solitary, soft, mobile swelling measuring approximately 1 × 1 cm was noted over the proximal phalanx (Figure 1a). The overlying skin appeared normal, with no signs of inflammation or neurovascular compromise. A clinical diagnosis of sebaceous cyst or benign soft tissue lesion was considered.

Radiological investigations were not performed, as the lesion was small, superficial, clinically benign-appearing, and showed no features suggestive of bony involvement. Given its accessibility and mobility, complete excision under local anesthesia was performed on 26-01-2025 at the All India Institute of Medical Sciences, Bibinagar. Intraoperatively, the lesion was well-circumscribed and not attached to adjacent structures or bone (Figure 1b).

Histopathological findings

Gross examination showed a skin-covered soft tissue mass measuring 1.5 × 1.5 × 1.0 cm. The cut surface was solid and gray-white, without hemorrhage or necrosis. Microscopically, the lesion was an unencapsulated, multinodular tumor in the dermis. The nodules comprised spindle-shaped cells with wavy nuclei, moderate eosinophilic cytoplasm, and indistinct cell borders. Focal nuclear palisading was present. A thin rim of fibroblastic or perineurial-like cells encased each nodule (Figures 1c, 1d). No mitotic activity, necrosis, myxoid change, or inflammatory infiltrate was seen. These features were suggestive of a benign spindle cell tumor, with differentials including PS and plexiform neurofibroma (PNF).

Immunohistochemical findings

Immunohistochemistry showed strong, diffuse S-100 immunopositivity in the tumor cells, confirming Schwann cell origin and supporting the diagnosis of PS (Figure 1e).

Outcome

The postoperative course was uneventful. At the one-year follow-up, the patient remained asymptomatic, with no recurrence.

## Discussion

PS is a rare variant of schwannoma, constituting approximately 2-5% of all schwannomas [2,6]. It was first described as a distinct entity by Masson et al. in 1970 [6]. This tumor is characterized by a distinctive multinodular or plexiform growth pattern composed of Schwann cells [2,7]. PS can occur at any age, but most cases are diagnosed in the third or fourth decades of life. Although there is no definite sex predilection, some reports suggest a slight male predominance [7].

Typically, these tumors arise in superficial sites such as the dermis and subcutaneous tissue of the head, neck, and extremities [4,8]. Rarely, they may involve deep soft tissues, visceral organs, and unusual locations such as the oral cavity, breast, testis, uvea, small intestine, colon, and spine. Some unusual cases have also been reported associated with macrodactyly and trigger finger [1,2,9,10].

Clinically, PS usually presents as a slow-growing, painless, solitary nodule and is often less than 2 cm in size, as in our case, although larger lesions have been described [2,7]. Our case, arising in the finger, is rare and aligns with earlier reports of digital PSs [1].

Radiologically, MRI may help differentiate PSs from neurofibromas. Schwannomas often show a peripheral low-intensity rim on T2-weighted images due to the fibrous capsule, whereas this finding is less common in neurofibromas [6]. The “target sign,” with central low and peripheral high T2 signal intensity, may be seen in both, but distribution patterns differ [6].

Grossly, PS appears as a multinodular or lobulated mass resembling a “bag of worms.” The nodules are firm, gray-white to tan, and typically lack true encapsulation, as was seen in our case [2].

Histopathologically, PS is mainly composed of Antoni A areas--cell-rich regions with spindle cells, nuclear palisading, and occasional Verocay bodies. Antoni B areas, which are less cellular and more myxoid, may be present in small quantities [8,10]. The multinodular arrangement, superficial location, and strong palisading are characteristic features, all of which were observed in our case.

On immunohistochemistry, PS consistently shows strong and diffuse S-100 positivity, confirming Schwann cell origin. It may also express SOX10. CD56 and calretinin may be positive in a minority of cases [8]. Our case demonstrated diffuse strong S-100 positivity.

The main histological differential is PNF, which also has a multinodular appearance but contains a mixture of Schwann cells, fibroblasts, and axons. PNF shows only patchy S-100 positivity and is strongly associated with neurofibromatosis type 1 (NF1), with a risk of malignant transformation--features not seen in our patient [3].

Another differential is plexiform fibrohistiocytic tumor (PFHT), which typically occurs in children and young adults, often in the upper limbs. PFHT consists of fibroblastic and histiocytic components, is negative for S-100, and expresses CD68, CD163, and vimentin, which help distinguish it from PS [11].

Both malignant peripheral nerve sheath tumors (MPNSTs) and PNFs may appear similar on radiological imaging, so histopathology is essential for differentiation. MPNSTs are infiltrative, highly cellular, and composed of elongated cells with pleomorphic nuclei, significant mitotic activity, and necrosis. In contrast, PSs are circumscribed and lack such aggressive features. Although atypical variants of PS may show increased cellularity and mitosis, unlike MPNST, they retain strong S-100 expression [3].

Most PS cases are sporadic, but some are associated with neurofibromatosis type 2 (NF2) or schwannomatosis, particularly when multiple lesions are present. These syndromic cases may involve mutations in the NF2, SMARCB1, or LZTR1 genes [8]. INI1/SMARCB1 immunostaining may show mosaic loss in PS associated with schwannomatosis. Unlike PNFs, PS is not strongly associated with NF1, although some cases may occur with NF2 or schwannomatosis [3,4].

Surgical excision is the treatment of choice. In functionally critical areas like the hand, careful dissection is required to avoid nerve damage [1]. In our case, the lesion was excised completely without complications, and no recurrence was observed during follow-up.

## Conclusions

Plexiform schwannoma (PS) is a rare benign nerve sheath tumor with a distinctive multinodular growth pattern that can pose diagnostic challenges, especially in uncommon sites such as the digits. Our case highlights a digital plexiform schwannoma in the middle finger of a 38-year-old woman, which presented as a long-standing, small, slowly enlarging, and clinically benign-appearing lesion. In this case, the lack of radiological investigation emphasizes that careful clinical judgment, supplemented by complete excision and histopathological examination, remains the cornerstone for diagnosing accessible and superficial lesions. Histopathology and immunohistochemistry, particularly strong and diffuse S-100 positivity, are important in confirming the diagnosis and distinguishing PS from mimickers such as plexiform neurofibroma (PNF), plexiform fibrohistiocytic tumor (PFHT), and malignant peripheral nerve sheath tumor (MPNST). Recognizing these differences is essential to prevent misdiagnosis and overtreatment, as PS carries no risk of malignant transformation, unlike PNF.

Surgical excision with preservation of adjacent nerve structures remains the treatment of choice, with excellent prognosis and low recurrence risk when excised completely. Our case underscores the importance of including PS in the differential diagnosis of nodular soft tissue tumors of the hand. It also contributes to the limited but growing literature on digital PS, reinforcing that awareness of this entity allows pathologists and clinicians to arrive at the correct diagnosis and offer appropriate management.
